# Editorial: New challenges and future perspectives in autonomic neuroscience

**DOI:** 10.3389/fnins.2023.1271499

**Published:** 2023-08-23

**Authors:** Viola Salvini, Riccardo Accioli, Pietro Enea Lazzerini, Maurizio Acampa

**Affiliations:** ^1^Department of Medical Sciences, Surgery and Neurosciences, University of Siena, Siena, Italy; ^2^Stroke Unit, Department of Emergency-Urgency and Transplants, Azienda Ospedaliera Universitaria Senese, “Santa Maria alle Scotte” General-Hospital, Siena, Italy

**Keywords:** heart failure, autonomic nervous system, GLUT1, GABA_B_ receptor agonists, Pressure Pain Sensitivity, gangliopathy

Over the last decade, there has been growing interest and important developments in Autonomic Neuroscience research. In this Research Topic, an international selection of high-quality papers highlighted the latest advancements and the improvements in the research techniques, offering new insights into the role of autonomic nervous system (ANS) activity in regulating metabolic pathways and multiple systems such as cardiovascular, gastrointestinal, renal and immune system.

Regarding metabolism, an interesting study conducted by Faber et al. sheds light on the role of the autonomic nervous system in glucose metabolism. Recent independent research has also shown that Pressure Pain Sensitivity (PPS), a parameter that measures the maximum pain and discomfort sensation at the chest bone, in response to a gradually increased pressure during a period of 3–5 s, could serve as an indicator of ANS activity, particularly reflecting the level of ANS disruption (Faber et al., 2021). Considering the significance of the ANS in regulating glucose levels, especially in relation to HbA1c values that mirror the glycaemic control status over the previous 2–3 months (Kohzuma et al., 2021), the use of PPS bears paramount clinical importance. It can serve as both as a diagnostic tool to assess ANS activity and as a potential therapeutic target in managing metabolic disorders.

The impact of ANS on metabolism holds great potential in the context of cardiovascular disease, both indirectly, as seen before in the correction of main risk factors such as diabetes, and directly, by influencing heart rate. Kaminosono et al. introduced an innovative approach to heart rate control, using optogenetic cardiac pacing as an alternative to conventional methods like electrical stimulation or drug administration. Their animal models provided compelling evidence linking light-induced myocardial contraction with vital functions such as blood flow and respiration rhythm, leading to heart rate recovery in bradycardic mice. This groundbreaking method could pave the way for less invasive pacemaker development that eliminates the need for pacing leads. Patients with type II diabetes and high cardiovascular risk are often prone to the development of kidney disease (Wanner et al., 2016) and renal congestion similar to heart failure (HF) patients (Seo et al., 2020). The assessment of left and right doppler-derived intrarenal venous flow (IRVF) has emerged as a valuable biomarker to evaluate renal circulation and predict cardiovascular disease prognosis (Iida et al., 2016). Sodium-glucose cotransporter 2 inhibitors (SGLT2i) have demonstrated their efficacy in improving the prognosis of patients with HF with reduced ejection fraction (HFrEF) and slowing down renal disease development in type II diabetes patients (Packer et al., 2020). By evaluating IRVF and administering SGLT2i, clinicians can effectively guide decongestion therapy. Another promising approach is neuromodulation, a novel treatment for HF that utilizes vagus nerve stimulation (VNS) to produce multiple cardioprotective effects (Hadaya and Ardell, 2020). In this regard, the case report by Nagai et al. showed the positive impact of transcutaneous VNS (tVNS) and SGLT-2i administration on modifying IRVF in a 77-year-old patient with HF with preserved ejection fraction (HFpEF), resulting in reduced renal congestion for HFpEF patients.

Another interesting study by Johnson et al. aimed to search for reliable markers to distinguish sacral parasympathetic nerves from other extrinsic and intrinsic nerves in the human colon, in order to better comprehend their functional role and the clinical significance of their disruption. Bowel innervation can be divided into two components: a descending innervation, which typically involves the proximal part of the bowel, and an ascending innervation, which typically involves the distal part. In this context, the authors have found that ascending nerves can be distinguished in the colorectum of humans using glucose transporter type 1 (GLUT1) labeling combined with NF200. As regards the control of gastric function, the local GABA(γ-aminobutyric acid)ergic signaling in the dorsal vagal complex plays an essential role (Gillis et al., 2022). Less well-known is the role of the GABA_B_ receptor (Gillis et al., 2022). Injection of baclofen, a selective GABA_B_ receptor agonist in the dorsal motor nucleus of the vagus (DMV), increases gastric tone and motility. Still to be evaluated is the effect of baclofen on gastric motility in the nucleus of the solitary tract (NTS). In their study Bellusci et al. compared the activation of the GABA_B_ receptor on the NTS by microinjecting baclofen into the NTS, and monitoring intragastric pressure. They also compare its action to optogenetic activation of somatostatin (SST). The results of this study show that GABA_B_ receptors in the NTS significantly increase gastric motility and tone. Optogenetic stimulation *in vivo* and *in vitro* suggests that these baclofen-activated receptors suppress glutamatergic sensory vagal afferents in the NTS and also inhibit interneurons and inhibitory neurons projecting to the DMV, which, in turn, increase motility through a cholinergic excitatory pathway to the stomach.

An acquired autoimmune disorder of ANS is represented by autoimmune autonomic gangliopathy (AAG), characterized by autonomic dysfunction, including sympathetic and parasympathetic failure (Vernino et al., 2000). In their study Yamakawa et al. established a mouse model of AAG representing an autoimmune dysautonomia, associated with MHC class II, to understand the pathogenic mechanism and pathogenicity of nicotinic acetylcholine receptor (nAChR) antibodies. The amino acid sequence of mouse nAChRα3 protein was analyzed using an epitope prediction tool to predict possible MHC class II binding mouse nAChRα3 peptides. The authors provided evidence that active immunization of mice with nAChRα3 peptides causes autonomic dysfunction as similarly occurs in human AAG, suggesting a mechanism by which different MHC class II molecules could preferentially influence the nAChR-specific immune response, thus controlling the diversification of the autoantibody response.

In conclusion, this Research Topic has made remarkable contributions, significantly advancing our understanding of various aspects within Autonomic Neuroscience. These studies have shed light on intricate and complex interrelationships between different systems, offering crucial clinical implications for patient management. Moreover, the valuable insights and recommendations provided pave the way for further exploration in this rapidly evolving field.

## Author contributions

VS: Conceptualization, Writing—original draft, Writing—review and editing. RA: Conceptualization, Writing—original draft, Writing—review and editing. PL: Conceptualization, Writing—original draft, Writing—review and editing. MA: Conceptualization, Writing—original draft, Writing—review and editing.
